# Updated Evidence on the Epidemiology of Hepatitis C Virus in Hemodialysis

**DOI:** 10.3390/pathogens10091149

**Published:** 2021-09-07

**Authors:** Fabrizio Fabrizi, Roberta Cerutti, Piergiorgio Messa

**Affiliations:** 1 Department of Nephrology, Dialysis and Renal Transplantation, Fondazione IRCCS Cà Granda Ospedale Maggiore Policlinico, 20122 Milan, Italy; roberta.cerutti@policlinico.mi.it (R.C.); piergiorgio.messa@policlinico.mi.it (P.M.); 2 Department of Clinical Sciences and Community Health, University of Milan, 20137 Milan, Italy

**Keywords:** hepatitis C virus, hemodialysis, incidence, infection control practices, prevalence, transmission

## Abstract

Prevalence rates of HCV infection are decreasing in hemodialysis units of most developed countries; however, nosocomial transmission of HCV continues to occur in the hemodialysis setting, not only in the emerging world. According to the Dialysis Outcomes and Practice Patterns Study (DOPPS, 2012–2015), the prevalence of HCV among patients on regular hemodialysis was 9.9%; in incident patients, the frequency of HCV was approximately 5%. Outbreaks of HCV have been investigated by epidemiologic and phylogenetic data obtained by sequencing of the HCV genome; no single factor was retrieved as being associated with nosocomial transmission of HCV within hemodialysis units. Transmission of HCV within HD units can be prevented successfully by full compliance with infection control practices; also, antiviral treatment and serologic screening for anti-HCV can be useful in achieving this aim. Infection control practices in hemodialysis units include barrier precautions to prevent exposure to blood-borne pathogens and other procedures specific to the hemodialysis environment. Isolating HCV-infected hemodialysis patients or using dedicated dialysis machines for HCV-infected patients are not currently recommended; reuse of dialyzers of HCV-infected patients should be made, according to recent guidelines. Randomized controlled trials regarding the impact of isolation on the risk of transmission of HCV to hemodialysis patients have not been published to date. At least two studies showed complete elimination of de novo HCV within HD units by implementation of strict infection control practices without isolation practices. De novo HCV within hemodialysis units has been independently associated with facility HCV prevalence, dialysis vintage, and low staff-to-patient ratio. Antiviral treatment of HCV-infected patients on hemodialysis should not replace the implementation of barrier precautions and other routine hemodialysis unit procedures.

## 1. Introduction 

Liver damage is relatively common in patients with chronic kidney disease, particularly those on regular dialysis and kidney transplant recipients. Chronic liver disease is an important cause of morbidity and mortality in these patients [1], and hepatitis B and C viruses remain important agents of liver damage in chronic kidney disease [2,3]. Some surveys have reported a reduction in the prevalence and incidence of HCV infection within dialysis units, but the extent of ongoing transmission of HCV between patients on regular hemodialysis is still unclear. The epidemiology of HCV in the hemodialysis population remains a hot topic, and various approaches have been adopted to prevent the spread of HCV, with controversial results. The aim of this narrative review is to give updated information on epidemiology and risk factors for hepatitis C virus in the dialysis population. 

## 2. Epidemiology of HCV Infection in Dialysis Population and Recent Evidence

After its identification in 1989, it has been discovered that patients on renal replacement therapy have frequently detectable anti-HCV antibody in serum. The frequency of patients with positive serology for anti-HCV antibody who undergo maintenance dialysis is high and currently ranges between 2% and 80% all over the world [3]. Infection with chronic HCV infection leads to chronic liver disease, with its attendant complications (cirrhosis, hepatocellular carcinoma, and hepatocellular failure). The prevalence rates of HCV infection in the dialysis population are greater than those reported among patients with intact kidneys, and this occurs in either developing or developed countries. A large body of studies was published in the 1990s regarding epidemiology and risk factors for HCV infection among patients undergoing hemodialysis, particularly in the developed world [4]. Since the early 2000s, the literature on this point has been less abundant, despite the ongoing transmission of HCV within dialysis units worldwide. 

The Dialysis Outcomes and Practice Patterns Study (DOPPS, 1996–2015) was performed to give a deeper insight on this subject [3]. According to the latest survey from the DOPPS (Dialysis Outcomes and Practice Patterns Study, 2012–2015), the prevalence of anti-HCV antibody in patients on HD in the DOPPS was 9.9% overall (21 countries globally). The prevalence of anti-HCV antibody decreased during the past 15 years in many countries, including Italy (dropped from around 23% to 12%), the US (dropped from around 11% to 7%), and Japan (dropped from around 19% to 12%). In other countries, such as Germany and the UK, the prevalence remained stable. 

The DOPPS investigators found 946 patients on HD with seroconversion for anti-HCV antibody among 37,995 initially seronegative patients [3]. Accordingly, the investigators at DOPPS extrapolated an annual incidence of 20,000 new cases of HCV acquired while on HD (global population on HD, *n* = 1,500,000 individuals). The HCV incidence (i.e., seroconversion for anti-HCV antibody among patients with an initial negative HCV antibody measurement) was 1.2 per 100 patient years in DOPPS 2 (2012–2015) and ranged from 0% (Belgium, Sweden, Turkey) to 2.9% in Italy. The most important predictors of HCV infection were facility HCV prevalence (HR, 1.26, 1.14–1.39 per 10% greater facility prevalence of HCV) and HBV (HR, 2.87; 95% CI, 2.06–4.00) and HIV infection (HR, 2.93; 95% CI, 1.79–4.80) [3]. 

Outbreaks of HCV among dialysis patients continue to occur all over the world; in their 2016 report, the CDC reported the occurrence of 36 patients with acute HCV in 19 hemodialysis facilities (eight states) between the 2014 and 2015 [5]. It is possible that some cases of de novo HCV that occurred in various clinical settings (including hemodialysis) were not reported to the CDC [5].

The information on prevalence and incidence rates of HCV among patients on regular dialysis in the emerging world is limited, and the most important survey to date has been given by the Asia-Pacific Dialysis Registry (*n* = 201,590), which found that the prevalence of positive anti-HCV serologic status ranged between 0.7% and 18.1% across 10 various areas and regions [6]. Numerous single-center surveys from emerging countries have been published in the last decade, and these have reported high prevalence and incidence rates (Table 1) [7,8,9,10,11,12,13,14,15,16,17,18,19,20,21,22,23,24,25,26,27,28].

## 3. Post-Transfusion Transmission of HCV within Dialysis Units

The most frequent transmission route of HCV infection occurs by percutaneous exposure to blood. Transfusions before screening of blood donors for anti-HCV antibody led to many cases of HCV in the dialysis setting in the 1990s. The most important risk factor for transmission of HCV in the US is injection drug use, which is responsible for the HCV increase since 2004. Post-transfusion HCV infection has been eliminated in the developed world as blood transfusions are currently safe in the US and European countries, with a residual risk of transmission of HCV lower than 1 per 1 million blood units. After the elimination of post-transfusion HCV infection, a subsequent decline in HCV incidence and prevalence within dialysis units in developed countries occurred. In addition to the screening of blood or blood products for HCV, the frequency of post-transfusion HCV lowered in the 1990s due a reduction in blood transfusion requirements following the introduction of erythropoietin-stimulating agents (ESAs) in the routine management of patients on regular dialysis [29].

## 4. Nosocomial Transmission of HCV within Dialysis Units (Infection Control Practices)

Blood transfusions are currently very safe in the developed world; however, the prevalence rates of HCV infection remain higher in patients on maintenance hemodialysis than in the respective general population; this is likely related to nosocomial transmission of HCV within hemodialysis units. Patients on hemodialysis are commonly anemic at baseline due to uremia, and blood transfusions had supported the spread of HCV among patients on hemodialysis. In addition to the post-transfusion route, nosocomial transmission of HCV occurs, and circumstantial evidence highlights this: (1) the independent relationship between dialysis vintage and prevalence of serum anti-HCV antibody, (2) the association between prevalence and incidence of HCV infection in single HD units, (3) the great homogeneity of HCV genotypes among HCV-infected patients in individual hemodialysis centers, and (4) the greater frequency of anti-HCV antibody in patients on hemodialysis than in those on peritoneal dialysis or home-HD treatment. After the elimination of post-transfusion HCV, (5) the incidence of HCV infection lowered but did not disappear, and this confirms again the nosocomial transmission of HCV infection [29]. 

The low adherence to infection control procedures against blood-borne pathogen agents remains the most important source of nosocomial transmission of HCV within hemodialysis units. These include “universal or standard precautions”: hand washing after contact with body fluids, glove-wearing when touching blood or other potentially infectious material, and wearing of face shields, gowns, and glasses when exposure to blood and body fluids is expected. 

**Figure 1:**
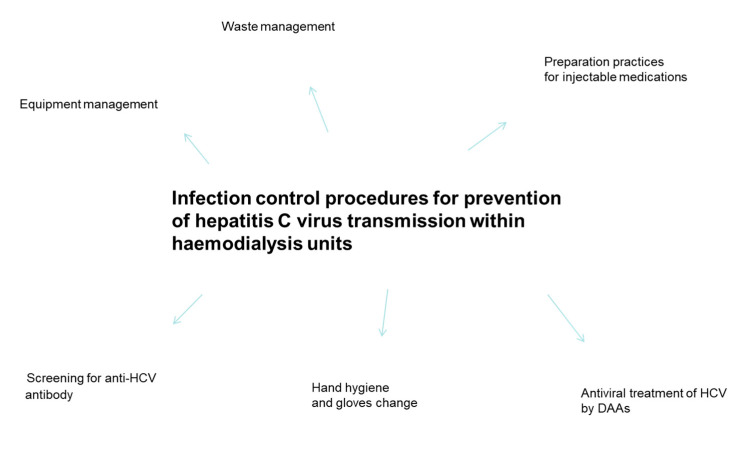
Overview of infection control practices for preventing HCV infection within hemodialysis units.

In addition to general precautions reported above, further precautions that are unique to the HD setting have been recommended: clear separation within HD units between clean and contaminated areas, where handling and storage of medications or handling of blood specimens and dialysis equipment after use is indicated, respectively [30]. Cleaning and disinfecting non-disposable items, environmental surfaces, and dialysis machines between uses has been suggested. Additionally, it has been recommended to avoid sharing of supplies, instruments, or medications between any patients, including ancillary supplement items (trays, clamps, scissors, blood pressure cuffs, and other non-disposable equipment components) [30] (Figure 1).

Nosocomial transmission of HCV has been confirmed by phylogenetic analysis, which offers the possibility of identifying clusters of closely related isolates of HCV; some portions of the HCV genome such as hypervariable region HCV 1 are very variable and can be used for fingerprinting of isolates or quasi-species using nucleic acid sequencing. This approach is analogous to the sequencing of the V3 region used in studies on the transmission of human immunodeficiency virus infection. Molecular virology tracing has been undertaken to investigate outbreaks of HCV infection among patients on regular dialysis or with intact kidneys. A total of 8 genotypes and 67 subtypes have been identified, and at least 10 viral proteins are produced in the life cycle of the virus: structural (core, envelope glycoproteins E1 and E2) and non-structural (ion channel p7, and NS2, NS3, NS4A, NS4B, NS5A, and NS5B). The NS5B region is most important for HCV genotyping and subtyping as well as for phylogenetic analysis. A few systematic reviews of studies adopting molecular virology to address outbreaks of HCV infection within HD units have already been published [31,32], and at least 25 reports have been retrieved. 

## 5. Nosocomial Transmission of HCV within Dialysis Units (Evidence Based on Systematic Reviews)

The most important mode of nosocomial transmission of HCV appears to be cross-contamination from supplies and surfaces (including gloves) due to lapses of infection-control procedures within hemodialysis units. Additional (and less important) transmission routes are direct contact between patients, invasive procedures outside HD setting using contaminated instruments, or dialysis during travel to developing countries.

Many outbreaks that occurred within dialysis units have been addressed with epidemiologic investigations and phylogenetic analysis. Epidemiologic investigations included interviews of patients or staff attending the dialysis facility, review of patient charts, and observation of infection control practices performed within the facilities. Patients on regular dialysis with de novo HCV frequently underwent dialysis in the same shift with other infected patients or shared the same dialysis machine during consecutive shifts [33].

Some systematic reviews have addressed the mechanisms of transmission of HCV within hemodialysis units. Multiple retrospective investigations aimed at understanding the exact cause of HCV acquisition within dialysis units have been published; these did not give clear evidence. Observational analyses of the HCV-associated outbreaks identified various possible modes of transmission of HCV but none were definitively cited as the source of the spread of HCV. Some explanations have been mentioned—the long latency of HCV infection, the number of dialysis sessions performed during the exposure period (up to three HD sessions per week), and the scarce documentation from dialysis medical records. These systematic reviews concluded that the occurrence of lapses in infection control procedures were associated with transmission of HCV between patients in dialysis units. According to a systematic review of reports on outbreaks (36 papers reporting on 45 outbreaks involving 335 unique patients on maintenance HD), sharing contaminated HD machines and multi-dose vials (heparin or saline solution) was the source of HCV transmission in some outbreaks (*n* = 14/45, 31%), while breaches in environmental cleaning and disinfection practices, failures in medication preparation and administration activities were the source in other outbreaks (*n* = 29/45, 64%) [31].

## 6. Nosocomial Transmission of HCV within Dialysis Units’ Isolation of HCV Positive Patients) 

The complete isolation of HBsAg positive carriers on maintenance hemodialysis (i.e., by dialysis rooms, staff, machines, and other equipment) has dramatically lowered the frequency of HBV within hemodialysis units. In addition, HBV and HCV share parenteral transmission, and patients on regular hemodialysis remain a group with high risk for parenteral infections. These findings initially suggested the isolation of HCV infected patients to control the spread of HCV infection within HD units. However, the evidence gained in subsequent years did not confirm this opinion, and isolation of HCV-infected patients to control HCV infection in the hemodialysis setting is now not recommended by numerous investigators and regulatory agencies. 

The CDC does not currently suggest designated machines or patient isolation to prevent nosocomial transmission of HCV within HD units; additionally, the CDC did not ban dialyzer reuse in the case of full adherence to standard infection control procedures. Two large prospective multicenter studies concluded that isolation does not provide protection against the risk of transmission of HCV between patients on maintenance hemodialysis [34,35]. In a longitudinal analysis from Italy, 2360 patients on maintenance hemodialysis (*n* = 58 dialysis units) were followed up for 1 year. Twenty-three patients showed seroconversion for anti-HCV antibody during the study period; according to the logistic regression analysis, the incidence of HCV infection was greater in units provided with a low personnel-to-patient ratio (OR, 5.4, 95%CI, 1.4–19.9) and those with a higher prevalence of HCV at baseline (OR, 4.6 95% CI, 1.4–15) [35].

Some authors were able to reduce the transmission of HCV within dialysis units after adoption of isolation policies of HCV-infected patients. Many of these studies compared their results with historical controls or adopted a “before-and-after” design. It remained unclear whether the positive results offered by these investigators resulted from isolation strategies per se or reinforced compliance with infection control practices. A Belgian cohort study was able to reduce the incidence of anti-HCV conversion to 0% after adoption of standard infection control practices [36]; another smaller study from Italy returned identical results [37]. 

Additional arguments that do not support the isolation of HCV-infected patients include the infectivity of HCV, which is lower than that of HBV. The adoption of isolation to prevent transmission of HCV includes the possibility of increased risk of HCV infection with more than 1 genotype. The cross immunity between viral strains is usual for HBV only, and the immunity acquired against an HCV strain offers little or no protection at all against infection by another HCV isolate. Even the time between acquisition of HCV and seroconversion for anti-HCV antibody hampers the policy of isolation of HCV-infected patients. The time between acquisition and seroconversion has been calculated to be a median time length of 5 months in patients on regular hemodialysis who undergo serological testing with third-generation ELISA; this is different from what happens for the HBV virus, where the monthly screening for HBV allows a prompt identification of de novo HBV.

## 7. Nosocomial Transmission of HCV within Dialysis Units (Dedicated Machines for HCV Positive Patients) 

Various investigators have suggested that HCV is transmitted through internal pathways of single-pass dialysis machines. It has been mentioned that HCV virions pass through the dialyzer membrane of an infected patient with migration to the fresh dialysate circuit, and that consequently, the presence of back-filtration across the dialyzer membrane allows the passage of HCV into the blood compartment of the second patient [38]. Some systematic reviews of the scientific literature based on molecular virology papers suggested that the internal contamination of HD machines plays a minor role, at most, in the nosocomial transmission of HCV among patients on maintenance hemodialysis. It is unlikely that the occurrence of a publication bias that suppresses the reporting of nosocomial transmission of HCV via internal fluid pathways of the dialysis machine leads to reports of transmission due to breaches in infection control procedures. 

According to a recent report from the Cochrane Database of Systematic Reviews, no RCTs have been conducted to compare the HCV incidence in dialysis units isolating HCV positive patients versus those units that did not [39]. One RCT regarded the strategy of dedicated HD machines for HCV-infected patients and the risk of transmission of HCV to patients on hemodialysis. It was a multicenter study enrolling a total of 12 HD units (*n* = 593 patients) assigned either to dedicated machines for HCV-infected patients or to machines that were not dedicated for the HCV-infected. The survey found that the incidence of HCV infection during the first follow-up period was 1.6% in the dedicated group and 4.7% in the control group (RR, 0.34, 95% CI, 0.11–1.07). During the second follow-up phase, the incidence was 1.3% in the dedicated group and 5.8% in the control group (RR, 0.22, 95% CI, 0.05–1.02) [40]. One of the participating units was excluded from the study due to poor adherence to the body fluid precautions for HD recommended by the CDC. The Cochrane reviewers concluded that no significant difference in the incidence of HCV infection between the two groups was observed. Of note, the quality of evidence was evaluated as “very low”, as the study design was biased in several points.

The 2018 KDIGO HCV Study Group recommended not using dedicated dialysis machines for patients with HCV infection and not isolating patients on regular hemodialysis who had HCV infection [41]. The same study group suggested that dialyzers from HCV-infected patients could be reused in the case of full adherence to standard infection control measures [41]. The adoption of isolation of HCV-infected dialysis patients or the use of dedicated dialysis machines for patients with HCV could generate the feeling of low risk of nosocomial spread of HCV and consequently a reduced attention to standard infection control practices (Figure 1).

## 8. Nosocomial Transmission of HCV within Dialysis Units: The Sources 

Numerous sources of outbreaks of HCV infection within HD units have been mentioned in the scientific literature. Gilli and colleagues [42] emphasized that sharing of heparin vials was an important cause of transmission of HCV. Kokubo et al. [43] demonstrated that contaminated multidose vials of saline solutions were shared between patients in the HD setting. Okuda and coworkers [44] reported a lack of glove use by the dialysis staff during repositioning of dialysis needles during the HD procedures. Sartor et al. [45] observed the blood contamination of the venous pressure monitoring system during accidental backflows, and transmission of HCV could have happened if another blood backflow occurred thereafter. Niu and coworkers [46] noted the overload of transducer protectors by increased arterial pressure in the circuit, resulting in reflux of blood across the transducer protector to the inside of the dialysis machines. Arenas Jimenez et al. [47] reported that poor hand washing before and, less frequently, after activities involving a risk of nosocomial transmission was a significant cause of diffusion of HCV in the hemodialysis setting. Delarocque-Astagneau and coworkers [48] suggested that poor environmental disinfection played a pivotal role in the contamination of HCV within HD units. 

Shimokura and colleagues [49] were able to link specific patient care practices to higher rates of HCV prevalence; this is in contrast with other reports that relied on self-reports instead of direct observation. They enrolled 53 hemodialysis facilities in the United States (*n* = 2933 patients); HCV positivity was confirmed in 294 patients (overall prevalence, 9.9%). After adjusting for non-dialysis-related HCV risk factors, patient care practices that were independently associated with higher rates of HCV prevalence were reusing priming receptacles without disinfection (OR, 2.3; 95% CI, 1.4–3.9), handling blood specimens adjacent to medications and clean supplies (OR, 2.2; 95% CI, 1.3–3.6), and use of mobile carts to deliver injectable medications (OR, 1.7; 95% CI, 1.0–2.8). Many investigators of outbreaks of HCV in dialysis units reported multiple breaches in infection control procedures but were not able to quantify facility-related factors that could increase the risk of environmental blood contamination.

An incomplete disinfection of the external machine surfaces or other surfaces at the dialysis station have been implicated in the development of numerous dialysis outbreaks; in fact, receiving dialysis next to an HCV-infected patient has been noted to be a risk factor for nosocomial spread of HCV infection [50]. An inappropriate disinfection and cleaning of the external surfaces of dialysis stations (at the end of each shift) is unfortunately a frequent occurrence in dialysis units, especially in overcrowded ones.

## 9. Nosocomial Transmission of HCV within Dialysis Units: Others 

Additional measures beyond infection control practices have been recommended to prevent the transmission of HCV within dialysis units. Periodic screening for anti-HCV antibody plays a pivotal role in identifying transmission of the virus. The 2018 HCV KDIGO Study Group recommended screening all patients for HCV upon initiation of hemodialysis or peritoneal dialysis [41]. Anti-HCV testing should be performed two times a year, and hemodialysis patients should be tested for ALT levels monthly. It is clear that the detection of seroconversion for anti-HCV antibody should require prompt notification of anti-HCV seroconversion and immediate actions by the staff. An audit of the adherence level to infection control procedures in the hemodialysis setting should be performed. Screening for anti-HCV antibody should not be considered an alternative to the implementation of infection control precautions.

The advent of DAAs has dramatically changed the management of HCV, even in patients with advanced chronic kidney disease. IFN-based therapy had been the cornerstone of antiviral therapy for HCV until a few years ago; however, IFN-based regimens have limited efficacy and safety in advanced kidney disease. Patients with CKD stage 4 or 5 and HCV infection have been historically considered a “difficult-to-treat” patient group, as have patients with HBV/HCV or HIV/HCV co-infection, among others. The goal of DAAs is to obtain the “sustained viral response”, in other words, a clearance of the virus that persists for at least 12 weeks after the end of antiviral therapy. Patients seropositive for anti-HCV antibody but without detectable HCV RNA in serum are without infectivity; thus, the treatment of HCV-infected patients should support the prevention of HCV transmission within dialysis units [41]. It is noteworthy that the first goal of treatment of DAAs use is the “cure” of patients and their consequent benefit. The transmission of HCV has even been reported in contexts with low prevalence rates of HCV; thus, full compliance with infection control strategies is again recommended. Treatment of HCV with DAAs cannot replace the other methods for preventing the transmission of HCV within dialysis units [41].

## 10. Conclusions

The frequency of HCV infection is decreasing within dialysis units in many high-income countries. However, prevalence and incidence rates remain high in the emerging world. Nosocomial transmission of HCV is currently the most important source of HCV spread within HD units all over the world. Infection control procedures recommended against the spread of HCV do not include isolation of HCV-infected patients (by dialysis rooms, machines, or staff). Various specific-care practices have been linked to higher rates of prevalence of HCV, including handling blood specimens adjacent to clean areas or using mobile carts to deliver injectable medications. Globally, we need to remove the barriers to the implementation of infection control practices within dialysis units. Regular screening for anti-HCV antibody, routine audits of infection control precautions, and focused training of dialysis staff continue to have primary importance in preventing transmission of HCV in dialysis units.

## Figures and Tables

**Table 1:** Prevalence rates of HCV infection among patients on hemodialysis in emerging world.

Authors	Prevalence	Country	Reference Year	Testing
Santana et al.	23.8% (94/395)	Bahia (Brazil)	2001	Serological
Othman et al.	25.7% (71/276)	Tunisia	2004	Molecular
Moukeh et al.	54.5% (299/550)	Syria	2006	Serological
Kopec et al.	18.6% (40/215)	Poland	2010	Serological
Alashek et al.	32.3% (769/2382)	Libya	2012	Serological
Ummate et al.	15% (15/100)	Maiduguri (Nigeria)	2014	Serological
Seck et al.	5.7% (6/106)	Senegal	2014	Serological
Daglar et al.	12.0% (24/201)	Turkey	2014	Molecular
Schiller et al.	32.3% (194/600)	Romania	2015	Serological
Lioussfi et al.	59.7% (40/67)	Morocco	2016	Serological
Rinonce et al.	80.7% (130/161)	Yogyakarta (Indonesia)	2016	Serological
Abou-Rached et al.	4.6% (177/3769)	Lebanon	2016	Serological
Luma et al.	19.2% (20/104)	Cameroon	2017	Serological
Jakupi et al.	52.9% (354/668)	Kosovo	2018	Serological
Kataruka et al.	9.7% (20/206)	India	2019	Molecular
Lodhi et al.	45.7% (54/118)	Quetta (Pakistan)	2019	Serological
Ali et al.	19.5% (94/480)	Pakistan	2019	Molecular
Duong et al.	12.9% (26/201)	Vietnam	2019	Molecular
Elmowafy et al.	47.9% (46/96)	Egypt	2019	Serological
Madhavan et al.	8% (8/100)	Kerala (India)	2020	Molecular
Mahupe et al.	1.7% (3/168)	Gaborone (Botswana)	2021	Serological
Kalita et al.	26% (51/196)	Rishikesh (India)	2021	Molecular

## Data Availability

Not applicable.

## Abbreviations

AE	Adverse events
AH	Arterial hypertension
AKI	Acute kidney injury
CDC	Centers for Disease Control and Prevention
CI	Confidence intervals
CKD	Chronic kidney disease
DAAs	Direct-acting antiviral agents
DM	Diabetes mellitus
ESA	Erythropoietin-stimulating agents
ESRD	End-stage renal disease
HBV	Hepatitis B virus
HCV	Hepatitis C virus
HIV	Human immunodeficiency virus
HR	Hazard ratio
HD	Hemodialysis
KDIGO	Kidney Disease: Improving Global Outcomes
NA	Not available
OR	Odds ratio
